# Superimposed Takotsubo syndrome and acute myocardial infarction: a systematic review and pooled analysis of published cases

**DOI:** 10.1016/j.ijcha.2026.101954

**Published:** 2026-06-11

**Authors:** Vladimir E. Shlyakhover, Zach Rozenbaum, Sharon Bruoha

**Affiliations:** Department of Cardiology, Barzilai University Medical Center and Faculty of Health Sciences, Ben-Gurion University of the Negev, Hahistadrut 2, Ashkelon, Israel

**Keywords:** Takotsubo syndrome, Acute myocardial infarction, STAMI

## Abstract

Takotsubo syndrome (TTS) has traditionally been regarded as a diagnosis of exclusion requiring the absence of obstructive coronary artery disease. However, increasing evidence suggests that acute myocardial infarction (AMI) and TTS may coexist as a combined overlap phenotype. We refer to this entity as STAMI (Superimposed Takotsubo and Acute Myocardial Infarction). This review summarizes currently available evidence regarding the clinical characteristics, imaging findings, mechanisms, and outcomes of this underrecognized syndrome. A systematic review of published case reports and case series identified 48 patients with simultaneous AMI and TTS reported between 2000 and 2025. The mean age was 67 years and 69% were women. STEMI was present in 57% and NSTEMI in 41% of cases. Although atherosclerotic plaque rupture was the predominant infarct mechanism, vasospasm and spontaneous coronary artery dissection accounted for more than 20% of presentations. A consistent finding across reports was an anatomical-functional mismatch in which wall-motion abnormalities extended beyond the culprit coronary territory, most commonly manifesting as apical ballooning. Marked QTc prolongation and disproportionately elevated natriuretic peptide levels were recurrent features. Initial left ventricular dysfunction was often severe but demonstrated substantial recovery during follow-up, supporting the presence of reversible stress-induced myocardial stunning in addition to ischemic injury. Cardiogenic shock occurred in approximately 10% of patients, while in-hospital mortality was 6.3%. Recognition of STAMI is clinically important because ventricular dysfunction frequently exceeds the expected infarct territory and may influence diagnostic interpretation, risk stratification, and therapeutic management strategies.

## Graphical abstract

**Figure:**
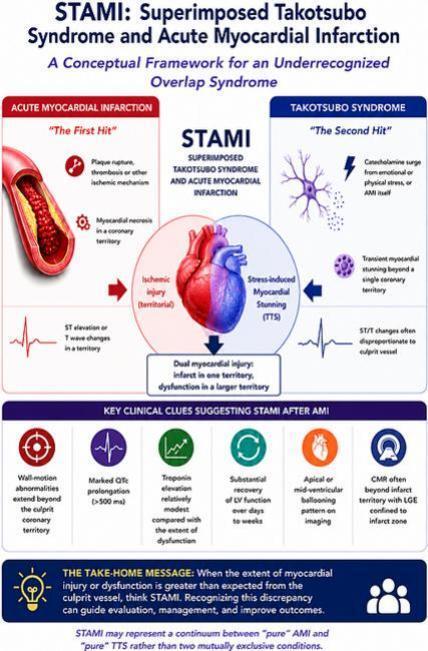

## 1 Introduction

Takotsubo syndrome (TTS) is frequently preceded by emotional [1], [2] or physical [3] stressors and has historically been considered a diagnosis of exclusion requiring the exclusion of obstructive coronary artery disease (CAD) [4]. This concept emerged from the marked clinical and electrocardiographic overlap between TTS and acute myocardial infarction (AMI). Traditionally, once a culprit coronary lesion was identified during angiography, left ventricular (LV) dysfunction was attributed solely to ischemic injury. However, growing evidence challenges this binary framework.

Recent data from the International Takotsubo Registry (InterTAK) demonstrated that concomitant CAD is not uncommon among patients with TTS [5]. Furthermore, published case reports increasingly describe simultaneous AMI and TTS occurring as a combined phenotype [6]. We refer to this entity as STAMI (Superimposed Takotsubo and Acute Myocardial Infarction). Rather than representing mutually exclusive diagnoses, AMI and TTS may coexist as interacting pathophysiological processes. Depending on the clinical scenario, either condition may serve as the initiating event, while the second process amplifies overall myocardial injury.

Recognition of STAMI has important clinical implications. Severe LV dysfunction extending beyond the culprit vessel territory may be incorrectly attributed solely to infarction. As a result, clinicians may overlook the stress cardiomyopathy component and initiate aggressive vasoactive therapy that could potentially worsen myocardial stunning [7]. The present review summarizes available evidence regarding STAMI and proposes a practical conceptual framework for recognizing this underdiagnosed overlap syndrome.

## 2 Methods

### 2.1 Study design

This study was designed as a systematic review with pooled descriptive analysis of published case reports and case series describing the simultaneous occurrence of AMI and TTS, referred to in this review as STAMI. The primary objective was to identify recurrent clinical, electrocardiographic, echocardiographic, and biomarker patterns associated with this overlap phenotype and to explore potential mechanisms linking ischemic myocardial injury with stress-induced cardiomyopathy.

### 2.2 Search strategy and data sources

We performed a comprehensive literature search to identify relevant reports published between January 1, 2000, and March 2025. Searches were conducted using publicly accessible electronic databases including PubMed/MEDLINE, Google Scholar, Semantic Scholar, and journal-specific search engines including Elsevier, Wiley, Springer, MDPI, and Cureus databases. Additional references were identified through manual cross-referencing of eligible reports.

Search terms included combinations of controlled vocabulary and free-text terms related to TTS and AMI. Boolean combinations included: “Takotsubo syndrome” OR “stress cardiomyopathy” AND “acute myocardial infarction” OR “STEMI” OR “NSTEMI” AND “simultaneous” OR “concomitant” OR “coexistence” AND “case report” OR “case series”.

### 2.3 Study selection

**Fig. 1:**
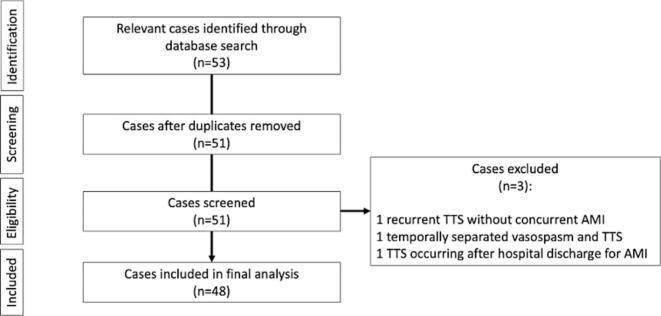
PRISMA flow chart.

We identified 53 potentially relevant clinical cases describing concomitant AMI and TTS. Following removal of 2 duplicate publications, 51 unique reports underwent full-text review. Three reports were subsequently excluded because they described recurrent TTS without concurrent AMI, temporally separate vasospasm and TTS (i.e., sequential rather than simultaneous events), or TTS occurring only after hospital discharge for AMI rather than as a concomitant presentation. A total of 48 cases met all predefined inclusion criteria and were included in the qualitative synthesis and pooled descriptive analysis [8], [9], [10], [11], [12], [13], [14], [15], [16], [17], [18], [19], [20], [21], [22], [23], [24], [25], [26], [27], [28], [29], [30], [31]. The review was conducted in accordance with PRISMA 2020 reporting principles for systematic reviews (Fig. 1).

### 2.4 Eligibility criteria

Eligible reports were required to include angiographic confirmation of coronary pathology consistent with AMI, including plaque rupture/thrombosis, spontaneous coronary artery dissection (SCAD), vasospasm, or coronary anomaly. In addition, reports were required to demonstrate imaging findings supporting concomitant TTS, including wall-motion abnormalities (WMA) extending beyond the expected vascular territory of the culprit lesion. Cases were excluded if clinical information was insufficient, if either diagnosis remained uncertain, or if the report described sequential rather than simultaneous pathology. Reports describing recurrent isolated TTS without concomitant AMI were also excluded. Duplicate reports were identified and removed.

### 2.5 Data extraction

Clinical data were extracted using a predefined standardized collection framework. Extracted variables included patient demographics, presenting symptoms, type of AMI [ST-segment elevation myocardial infarction (STEMI) vs. non-ST-segment elevation myocardial infarction (NSTEMI)], and, if available, QTc duration, time from symptom onset to revascularization, one vessel vs multivessel CAD, culprit coronary vessel [Left anterior descending (LAD), Left circumflex (LCX), Right coronary artery (RCA)], infarct mechanism (atherosclerosis/thrombosis, SCAD, vasospasm, anomaly), pattern of WMA and biomarker profiles (Table 1 and Table 2). Treatment strategies and clinical outcomes were also recorded.

**Table 1:** TTS diagnostic modalities.

Diagnostic Method	N	%	Role in STAMI Diagnosis
CAG	48	100%	Confirmation of an obstructive lesion (“culprit” artery), proving AMI
ECG	48	100%	Determination of AMI type (STEMI/NSTEMI) and identification of specific signs of superimposed Takotsubo: QTc prolongation >500 ms and giant inverted T-waves extending beyond the infarct zone
TTE	45	94%	Determination of LVEF and detection of mismatch: Identification of akinetic zones not corresponding to the territory of the infarct-related artery (e.g., apical ballooning in lateral AMI)
LVG	35	73%	Intraprocedural Imaging: Assessment of ventricular shape (apical ballooning) during catheterization
Biomarkers (Troponin/BNP)	15	31%	Dissociation: Identification of disproportionately high BNP levels relative to moderate troponin levels
CMR	11	23%	Tissue Differentiation: Simultaneous visualization of two pathologies: necrosis/scar by LGE from infarction and global edema from TTS
Intravascular Imaging (OCT or IVUS)	3	6%	AMI mechanism: Confirmation of SCAD or atherosclerosis in complex cases

TTS takotsubo syndrome; CAG coronary angiography; ECG electrocardiography; TTE transthoracic echocardiography; LVEF left ventricular ejection fraction; LVG left ventriculography; STAMI superimposed takotsubo and acute myocardial infarction; AMI acute myocardial infarction; STEMI ST-segment elevation myocardial infarction; NSTEMI non-ST-segment elevation myocardial infarction; BNP brain natriuretic peptide; CMR Cardiac magnetic resonance imaging; LGE late gadolinium enhancement; OCT optical coherence tomography; IVUS intravascular ultrasound; SCAD spontaneous coronary artery dissection.

**Table 2:** Patient demographics and Clinical characteristics.

Variable	n (%)
**Female**	33 (69%)
**Age (years ± SD)**	67.3 ± 13.8

**ECG at presentation**	
STEMI	27 (57%)
NSTEMI	20 (41%)
PEA	1 (2%)
QTc duration (ms)^	533 ± 63

**Time from pain onset to appropriate treatment**	
≥3 h	13 (27)
<3 h	7 (15)
Not reported	28 (58)

**Coronary vessels involved**	
Single vessel	36 (75%)
Multivessel	12 (25%)

**Culprit coronary vessel**	
LAD/Diagonal branches	27 (56%)
LCX	11 (23%)
RCA	6 (12%)
Not reported	4 (9%)

**Mechanism of myocardial infarction**	
Atherosclerosis	38 (79%)
Spasm	5 (11%)
SCAD	4 (8%)
Anomaly	1 (2%)

**Pattern of wall motion abnormalities**	
Apical	21 (45%)
Mid-Apical	19 (39%)
Mid ventricular	3 (6%)
Focal	2 (4%)
Biventricular	2 (4%)
Antero-lateral	1 (2%)

ECG electrocardiography; STEMI ST-segment elevation myocardial infarction; NSTEMI non-ST-segment elevation myocardial infarction; PEA pulseless electrical activity; QTc corrected QT interval duration; LAD left anterior descending; LCX left circumflex; RCA right coronary artery; SCAD spontaneous coronary artery dissection; ^ 10 cases reported the QTc interval.

WMA patterns were categorized as apical, mid-apical, mid-ventricular, focal, or biventricular (Table 1). Biomarker data included troponin I, troponin T, brain natriuretic peptide (BNP), and N-terminal BNP (NT-proBNP) values when reported (Table 2). Outcome variables included cardiogenic shock, LV thrombus formation, recovery of LV systolic function, and in-hospital mortality.

### 2.6 Data analysis

Because all included publications consisted of case reports or small case series, analyses were descriptive rather than meta-analytic. Continuous variables are presented as means with standard deviations, medians, or ranges as appropriate. Categorical variables are presented as frequencies and percentages. No formal weighting or quantitative meta-analysis was performed due to heterogeneity of reporting and variability in available clinical data.

## 3 Results

### 3.1 Diagnostic approaches

For STAMI identification, authors employed various combinations of diagnostic modalities depending on the publication year and available technology.

Three main diagnostic strategies emerged from the literature (Table 1). The classic invasive strategy combined coronary angiography (CAG) with left ventriculography (LVG). CAG established the diagnosis of AMI, while LVG demonstrated ballooning patterns extending beyond the culprit vessel territory. A second commonly used approach integrated CAG, transthoracic echocardiography (TTE), ECG, and biomarker analysis. TTE frequently revealed disproportionate WMA, whereas ECG demonstrated marked QTc prolongation and giant T-wave inversion. The most definitive diagnostic approach utilized cardiac magnetic resonance (CMR) imaging. CMR demonstrated focal late gadolinium enhancement (LGE) corresponding to infarct scar together with diffuse myocardial edema extending beyond the infarct territory, findings compatible with dual pathology.

These observations are consistent with the possibility that STAMI may represent a continuum between “pure” AMI and “pure” TTS rather than two mutually exclusive conditions.

### 3.2 Patient characteristics

A total of 48 cases meeting our predefined working criteria for STAMI were included. Mean age was 67.3 ± 13.8 years (range: 36–94 years) and women accounted for 69% of patients (*n* = 33).

### 3.3 Clinical characteristics

STEMI occurred in 57% and NSTEMI in 41%. Atherosclerotic plaque rupture represented the predominant infarct mechanism (79%), although vasospasm and SCAD together accounted for more than 20% of cases. The sex distribution differed from that typically observed in isolated AMI, which is predominantly male, and more closely resembles the female predominance of TTS. Electrocardiographically, marked QTc prolongation emerged as a recurring feature. In the case series by Christodoulidis et al., all four patients with AMI + TTS exhibited QTc prolongation (561 ms, 525 ms, 525 ms, 609 ms), which the authors identified as a marker of superimposed TTS^13^. In the case reported by Kundapur et al., QTc reached 642 ms^12^. González Alirangues et al. described “marked QTc prolongation” with dynamic evolutionary changes [14]. In total, 10 QTc measurements were identified across reports, averaging 533 ± 63.6 ms. In many cases, QT prolongation persisted despite successful reperfusion, suggesting ongoing catecholamine-mediated myocardial dysfunction.

Single-vessel disease was present in 75% of cases. This highlights that localized ischemia in the distribution of any of the three major coronary arteries may trigger global LV dysfunction (TTS) extending beyond the distribution of a single arterial territory. Multivessel disease was identified in 25% of cases (Table 2). This scenario complicates interpretation of WMA, as multi-segment akinesis may be attributed to multivessel CAD rather than to concomitant TTS. Therefore, the true incidence of STAMI may be underrecognized and likely exceeds that observed in isolated single-vessel ischemia.

There was a clear predilection for involvement of larger coronary arteries, with frequency correlating with vessel size: LAD (56%) > LCX (23%) > RCA (12%) (Table 2). The extensive anterior-apical ischemic burden associated with LAD-related AMI frequently masked the TTS ballooning component. However, even in LAD-related infarctions, segmental abnormalities commonly extended beyond the LAD distribution, involving basal or inferior segments. Likewise, patients with LCX or RCA occlusion frequently developed global apical ballooning, an anatomically implausible pattern for isolated infarction raising suspicion for a concomitant TTS component.

The most characteristic finding across reports was an anatomical-functional mismatch in which WMA extended beyond the territory supplied by the culprit artery. Apical ballooning represented the dominant phenotype (>80%), characterized by akinesia of the apical and mid-apical segments with basal hypercontractility (Table 1).

Biomarker patterns also demonstrated a hybrid phenotype (Table 3). Troponin elevation was often relatively modest compared with the severity and extent of ventricular dysfunction, whereas BNP and NT-proBNP levels were disproportionately elevated, reflecting extensive myocardial wall stress beyond localized infarction [32].

**Table 3:** Biomarkers in STAMI.

Biomarker	n	Range (Min - Max)	Arithmetic Mean	Geometric Mean	Log10 Mean ± SD
**Troponin I (ng/L)**	22	3–45,000	12,082	3411	3.53 ± 0.99
**Troponin T (ng/L)**	7	177–2164	706	528	2.72 ± 0.32
**BNP (pg/mL)**	7	13–35,000	5221	250	2.40 ± 1.03
**NT-proBNP (pg/mL)**	7	45–6730	2608	1147	3.06 ± 0.72

Given the extreme distribution of values, arithmetic means were disproportionally influenced by outliers. Accordingly, logarithmic transformation and geometric means provided a more accurate representation of the “typical” biomarker profile in STAMI; STAMI Superimposed Takotsubo and Acute Myocardial Infarction; BNP brain natriuretic peptide; NT-proBNP N-terminal BNP.

Initial LV systolic function, demonstrated on serial echocardiography and/or CMR was often severely depressed, with typical LV ejection fraction (LVEF) values ranging from 20% to 40%, reflecting the additive effects of infarction and global myocardial stunning. Despite profound dysfunction during the acute phase, substantial recovery of global ballooning and persistence of a localized WMA corresponding to the infarct territory were frequently noted during follow-up, consistent with a reversible stress-induced myocardial stunning component characteristic of TTS. Long-term follow-up typically demonstrated substantial improvement in LVEF, often normalizing to >50–55%, with only a small residual scar.

The extensive akinesia in STAMI predisposes to complications that are less common in small, uncomplicated infarctions. Cardiogenic shock occurred in approximately 10% of patients. LV thrombus formation was reported in 6.3%, likely related to extensive apical akinesia and blood stasis. In-hospital mortality was also 6.3%. Although limited by very small numbers, mortality appeared numerically higher among men compared with women (20% vs. 0%).

### 3.4 Revascularization and therapeutic management

Selection of revascularization strategy reflected the underlying mechanism of the infarction. Percutaneous Coronary Intervention (PCI) was performed according to standard practice in most cases (79%), primarily in the setting of atherosclerotic plaque rupture and thrombosis. Successful reperfusion abolished the ischemic trigger, whereas the TTS-mediated global dysfunction typically persisted during the early post-PCI period. In contrast, conservative therapy was favored in patients with SCAD, most commonly young-to-middle-aged women, due to the risk of dissection extension with stenting. Patients with vasospasm-induced infarction were treated with calcium-channel blockers and nitrates without stent implantation.

Given the severity of LV dysfunction, guideline-directed medical therapy for heart failure (ACE inhibitor/ARB, beta-blockers, diuretics) was universally prescribed. Anticoagulation was added when LV thrombus was present.

### 3.5 Clinical outcomes and prognosis

A defining feature of STAMI is the rapid and substantial improvement of contractility. LVEF at admission averaged 30–35%, reflecting the combined effects of infarction and stress-induced myocardial stunning. Follow-up assessments (5 days to 6 weeks) demonstrated recovery of LVEF to 50–60%. Complete recovery of global contractility was frequently accompanied by persistent localized hypokinesia or scar (LGE on CMR), confined to the infarct-related territory of the artery, whereas the Takotsubo ballooning segments recovered fully. Despite this favorable recovery of contractility, the initial “double hit” to the myocardium contributed to considerable early morbidity. The acute phase was often complicated by phenomena associated with extensive akinesia. LV apical thrombus was recorded in 3 cases (6.3%). Cardiogenic shock occurred in 5 patients (10%), attributed to the combined effects of necrosis and stunning, and required inotropic or mechanical circulatory support. Three in-hospital deaths were reported (6.3%). Although limited by very small numbers, mortality appeared numerically higher among men compared with women: 20% in men (3 of 15) versus 0% in women (0 of 33). Fatal cases occurred in elderly male with significant comorbidities. One presented with cardiogenic shock at admission, a feature known to confer high risk [33]. Whether this reflects biological susceptibility, delayed presentation, or comorbidity burden warrants further investigation.

## 4 Discussion

This pooled analysis supports the concept that AMI and TTS may coexist as a clinically meaningful overlap phenotype rather than represent mutually exclusive diagnoses. The most consistent finding across the reviewed reports was an anatomical-functional mismatch in which WMA extended well beyond the territory supplied by the culprit coronary artery. This observation was particularly striking in LCX- or RCA-related infarctions accompanied by diffuse apical ballooning but was also observed in many LAD infarctions, suggesting the presence of a superimposed stress-mediated myocardial stunning component.

The relationship between AMI and TTS is likely bidirectional. AMI may act as a major physical stressor capable of triggering secondary TTS through sympathetic overactivation and catecholamine excess. Severe pain, prolonged ischemia, hemodynamic instability, and emotional distress related to the acute coronary event may together contribute to diffuse transient myocardial dysfunction [6]. Conversely, primary TTS may contribute to myocardial ischemia through coronary vasospasm, microvascular dysfunction, increased myocardial oxygen demand, or marked elevations in left ventricular filling pressures. Therefore, STAMI should be viewed as a bidirectional overlap syndrome rather than a unidirectional disease process, with either pathology potentially serving as the initiating event [7].

Several recurring clinical features may help clinicians suspect STAMI in patients presenting with AMI (Table 4). Marked QTc prolongation, particularly when persistent despite successful reperfusion, emerged as a recurrent finding and may represent an important clue to a concomitant TTS component. Catecholamine-mediated oxidative stress and reactive oxygen species generation within cardiomyocytes may result in ion-channel dysfunction. These electrophysiological changes prolong ventricular repolarization and may explain the characteristic QTc prolongation and deep T-wave inversion frequently observed in TTS [7], [34], [35], [36]. In addition, severe LV dysfunction that appears disproportionate to infarct size, diffuse ballooning extending beyond the culprit territory, the characteristic edema on CMR [41], and disproportionate biomarker profiles collectively suggest the possibility of a superimposed stress cardiomyopathy component in at least a subset of patients. Alternative explanations for disproportionate WMA include myocardial stunning, multivessel ischemia, reperfusion injury, microvascular dysfunction, or anatomical coronary variation (wrap-around LAD) [37], [38], [39], [40].

**Table 4:** Proposed Clinical Clues Suggesting STAMI in Patients Presenting with AMI.

Feature	Findings Suggestive of STAMI	Clinical Significance
WMA	WMA extending beyond the expected vascular territory of the culprit artery	An important clue suggestive of superimposed stress cardiomyopathy
Apical ballooning in non-LAD infarction	Diffuse apical or mid-apical ballooning despite LCX or RCA culprit lesion	Anatomically difficult to explain by isolated infarction alone
Disproportionate LV dysfunction	Severe reduction in LVEF relative to infarct size or angiographic findings	Suggests additional catecholamine-mediated stunning
QTc prolongation	Marked QTc prolongation (>500 ms), especially when persistent after reperfusion	Characteristic electrical feature suggestive of concomitant TTS
Dynamic ECG evolution	Giant T-wave inversion and evolving QT prolongation after PCI/reperfusion	Typical of stress cardiomyopathy physiology
Biomarker dissociation	BNP/NT-proBNP disproportionately elevated relative to troponin levels	Reflects diffuse myocardial wall stress rather than focal necrosis alone
Rapid reversibility of WMA	Substantial improvement in ventricular function over days to weeks	Supports transient myocardial stunning component
CMR dual-tissue signature	Focal infarct-related LGE together with diffuse edema extending beyond infarct territory	Most supportive imaging evidence for dual pathology
Single-vessel AMI with diffuse dysfunction	Localized coronary occlusion accompanied by global or multi-territory dysfunction	Raises suspicion for secondary stress-mediated injury
Non-atherosclerotic AMI mechanisms	SCAD or coronary vasospasm associated with diffuse LV dysfunction	May represent potent triggers for stress cardiomyopathy
Clinical-hemodynamic disproportion	Severe shock or heart failure despite limited infarct burden	Suggests additive effect of ischemia and stress cardiomyopathy
Persistent dysfunction after successful PCI	Continued diffuse LV dysfunction despite restoration of coronary flow	Suggests ongoing catecholamine-mediated myocardial stunning

STAMI Superimposed Takotsubo and Acute Myocardial Infarction; AMI acute myocardial infarction; WMA Wall Motion Abnormality; LAD left anterior descending; LCX left circumflex; RCA right coronary artery; LV left ventricle; LVEF left ventricular ejection fraction; QTc corrected QT interval duration; TTS takotsubo syndrome; PCI percutaneous coronary intervention; BNP brain natriuretic peptide; NT-proBNP N-terminal BNP; CMR cardiac magnetic resonance imaging; LGE late gadolinium enhancement; SCAD spontaneous coronary artery dissection.

Another important feature is the characteristic reversibility of the WMA, suggestive of transient stress-induced myocardial stunning [35]. In many reported cases, severe diffuse LV dysfunction observed during the acute phase improved substantially within days to weeks, often leaving only a limited residual abnormality corresponding to the infarct-related territory.

Multimodality imaging appears central to the diagnosis of STAMI. Echocardiography and LVG frequently demonstrate WMA extending beyond the expected infarct territory, whereas CMR provides the strongest evidence for dual pathology by demonstrating focal infarct related LGE together with myocardial edema extending beyond the infarct zone. Intracoronary imaging such as optical coherence tomography and intravascular ultrasound may further improve diagnostic accuracy by identifying plaque rupture, plaque erosion [42], SCAD, or other coronary abnormalities not evident on angiography alone [43]. This is particularly relevant in patients presenting with features of myocardial infarction with non-obstructive coronary arteries or when the angiographic findings appear disproportionate to the degree of myocardial injury [44]. Greater use of intracoronary imaging in future studies may improve understanding of the underlying coronary substrate and refine the diagnosis of STAMI.

Recognition of STAMI may also have therapeutic implications. Identification of a superimposed TTS component may explain severe ventricular dysfunction that appears disproportionate to angiographic findings and should prompt careful screening for complications such as LV thrombus. Furthermore, in hemodynamically unstable patients, excessive catecholamine administration may theoretically aggravate myocardial stunning, whereas mechanical circulatory support may represent a safer strategy in selected cases [45]. Importantly, however, recognition of TTS should never delay timely reperfusion therapy when an acute culprit coronary lesion is identified.

Despite severe ventricular dysfunction during the acute phase, recovery of systolic function was common, consistent with the reversible nature of stress-induced myocardial stunning. Nevertheless, the overlapping ischemic and catecholamine-mediated injury was associated with substantial early morbidity, including cardiogenic shock, thrombus formation, and in-hospital mortality.

This study has several limitations. The analysis was based entirely on retrospective case reports and small case series and is therefore inherently subject to publication bias, reporting heterogeneity and incomplete clinical data. The limited use of systematic intracoronary imaging may have resulted in under recognition of mechanisms such as healed SCAD or other subtle coronary abnormalities. Furthermore, the true prevalence of STAMI is likely underestimated because concomitant TTS may remain unrecognized once obstructive coronary disease has been identified.

In conclusion, STAMI appears to represent a clinically meaningful overlap syndrome characterized by simultaneous ischemic and catecholamine-mediated myocardial injury. Greater awareness of this phenotype may improve diagnostic accuracy, facilitate recognition of disproportionate ventricular dysfunction after AMI, and influence management strategies in selected patients.

## Declaration of generative AI and AI-assisted technologies in the manuscript preparation process

During the preparation of this work the authors used ChatGPT based on the GPT-5.5 model in order to generate the Graphical abstract. After using this tool/service, the authors reviewed and edited the content as needed and take full responsibility for the content of the published article.

## CRediT authorship contribution statement

**Vladimir E. Shlyakhover:** Writing – review & editing, Writing – original draft, Visualization, Validation, Supervision, Software, Resources, Project administration, Methodology, Investigation, Formal analysis, Data curation, Conceptualization. **Zach Rozenbaum:** Validation, Supervision. **Sharon Bruoha:** Writing – review & editing, Writing – original draft, Visualization, Validation, Supervision, Software, Resources, Project administration, Methodology, Investigation, Formal analysis, Data curation, Conceptualization.

## Declaration of competing interest

The authors declare that they have no known competing financial interests or personal relationships that could have appeared to influence the work reported in this paper.
